# Practical tips in bronchiectasis for Primary Care

**DOI:** 10.1038/s41533-022-00297-5

**Published:** 2022-09-08

**Authors:** Miguel Angel Martinez-Garcia, Alberto Garcia-Ortega, Grace Oscullo

**Affiliations:** 1 Pneumology Department, Hospital Universitario y Politécnico la Fe de Valencia, Valencia, Spain; 2 CIBERES de enfermedades respiratorias, Instituto de salud Carlos III, Madrid, Spain

**Keywords:** Chronic obstructive pulmonary disease, Asthma

## Abstract

Bronchiectasis is the third most common chronic inflammatory airway disease, after chronic obstructive pulmonary disease (COPD) and asthma with a prevalence clearly underestimated probably because of its clinical similitudes with other chronic airway diseases. Bronchiectasis can be caused by a dozen of pulmonary and extra-pulmonary diseases and a variable number and severity of exacerbations can appear throughout its natural history, usually with an infectious profile. The dilation of the airway and the inflammation/infection is their radiological and pathophysiological hallmarks. Primary Care should play an important play in many aspects of the bronchiectasis assessment. In this article, we will try to offer a series of important concepts and practical tips on some key aspects of the diagnosis and management of bronchiectasis in Primary Care: clinical suspicion, diagnostic methods, severity assessment, overlap with asthma and COPD and microbiological and therapeutic aspects.

## Introduction

Asthma and chronic obstructive pulmonary disease (COPD) are the most common chronic inflammatory airway diseases. They have been estimated to affect more than 5–10% of the world population^1–5^. Although both asthma and COPD remain clearly underdiagnosed, it is very important to consider that they are not the only existing chronic inflammatory airway diseases that can be clinically suspected in Primary Care, and therefore a good differential diagnosis must be made with respect to other entities. It must be borne in mind that a diagnosis is not necessarily always going to be COPD (or only COPD) in a male smoker with airflow obstruction, or asthma (or only asthma) in a non-smoking female with airflow obstruction, even though these diagnoses are correct most of the time. It is always necessary to think that these same symptoms and signs can appear in other airway diseases, and if these are correctly diagnosed a patient’s follow-up and treatment may have to be altered.

Bronchiectasis is the third most common chronic inflammatory airway disease, after COPD and asthma. Its prevalence is not known, but it is estimated to be around 350–500 cases/100,000 inhabitants^6^ and it increases rapidly with age. Its symptoms can easily be confused with those of COPD and asthma^7^. Bronchiectasis can be caused by dozens of pulmonary and extra-pulmonary diseases (although in up to 30–50% of cases the cause is unknown)^8,9^ and, as in COPD and asthma, a variable number and severity of exacerbations can appear throughout its natural history, usually with an infectious profile^10^. A global consensus of experts recently defined bronchiectasis as a clinical-radiological disease consisting of dilation of the bronchial lumen accompanied by compatible symptoms (usually chronic cough plus expectoration with a purulent component)^11^. This dilation of the bronchial lumen is produced by a vicious circle of inflammation and chronic infection that injures the bronchial wall, producing irreversible destruction^12^. Bronchial inflammation and infection are therefore two key points in the management of these patients^13–15^, and, as in COPD and asthma, chronic airflow obstruction is their most frequent functional pattern^16^. In severe cases, systemic inflammation^17,18^ (predominantly neutrophilic^12^ but sometimes with a significant eosinophilic profile) is present^19–21^. In this article, we will try to offer a series of important concepts (Box 1) and practical tips (Box 2) on the diagnosis and management of bronchiectasis in Primary Care. However, it is important to highlight that these recommendations could vary according to the geographical area and the resources available in each respective setting.

Box 1 Important concepts for bronchiectasis in Primary Care

**Table:** 

Concept	Definition and reference
Bronchiectasis (BE)	Clinical + radiological condition
High-resolution computed tomography (HRCT scan)	CT scan with at least 1-mm slide of the pulmonary parenchyma. Diagnosis tool of choice for BE
*Pseudomona aeruginosa* (PA) infection	Most virulent microorganism in BE. When PA is isolated, it should be treated in case of frequent exacerbations.
Chronic bronchial infection (CBI)	Evidence of positive respiratory tract cultures of the same
	Microorganisms on two or more occasions at least 3 months apart over 1 year while in a stable state
Macrolides	These drugs have anti-inflammatory/immunomodulatory properties at low doses and over a prolonged period.
Inhaled antibiotics	Use in BE for the treatment of CBI infection by pathogenic microorganisms (especially PA) in the case of frequent exacerbations.
Overlap syndrome	Usually for defining patients with asthma + BE and COPD + BE.
Lung microbiome	Community of microorganisms that can be found living together in the lung.
Dysbiosis	Changes in the microbiome homoeostasis,

Box 2 Practical tips for bronchiectasis in Primary Care Clinical suspicion.

- A complete clinical history + lung function test + Chest X-ray + basic peripheral blood sample analysis are essential in Primary Care to evaluate the most probable aetiology of bronchiectasis
- Rule out bronchiectasis if there is:
  1. Presence of persistent and productive cough (especially with a haemoptoic or purulent component)
  2. Repeated isolation of pathogenic microorganisms, especially *Pseudomonas aeruginosa*
  3. Uncontrolled COPD or asthma patients, despite correct treatment
  4. Chronic respiratory symptoms and an underlying disease that could generate bronchiectasis.

Diagnostic method

- Bronchiectasis is not usually visible in a chest X-ray
- The diagnostic tool of choice is a high-resolution CT scan.

Overlap syndromes

- Bronchiectasis should not be confused with COPD and asthma in diagnosis
- COPD/asthma and bronchiectasis can coexist (overlap syndromes)
- Both disorders should be treated in overlap syndromes
- Special care with the use of ICs: use the lowest possible dose.

Microbiological aspects

- Serial sputum examination is important in bronchiectasis
- *Pseudomonas aeruginosa* is the most virulent microorganism
- The persistence of the same pathogenic microorganism is called chronic bronchial infection.

Therapeutic aspects

- Bronchiectasis patients should be managed on a multidisciplinary basis between Primary Care and secondary/tertiary care
- Sputum colour is the best biomarker for telling whether or not a given anti-inflammatory/antibiotic treatment is effective.
- Respiratory physiotherapy/rehabilitation and exercise programmes are key aspects of the treatment
- ICs should be avoided if asthma or COPD with eosinophilia is not present
- Macrolides are usually prescribed in bronchiectasis because of their immunomodulatory properties, rather than their antibiotic properties
- The control of comorbidities and end-stage patients is a key aspect of Primary Care in bronchiectasis
- Inhaled antibiotics currently provide effective treatment to some bronchiectasis patients
- Mild-to-moderate exacerbations should be treated in Primary Care
- The follow-up of post-COVID-19 patients is important since some of them develop traction bronchiectasis, among other pulmonary sequelae.
- Clear criteria for patients referral to secondary/tertiary centres

## Clinical suspicion

As has already been mentioned, it is of great importance that *the diagnosis of bronchiectasis should not be confused with that of COPD or asthma*. The presence of bronchiectasis must be ruled out, independently of any previous diagnosis of COPD or asthma, in any patient with: chronic respiratory symptoms and an underlying disease that causes bronchiectasis; persistent cough especially with usual expectoration (frequently with a purulent or haemoptoic component); chronic rhinosinusitis with productive cough; repeated sputum isolation of pathogenic microorganisms (especially *Pseudomomas aeruginosa* [PA]); uncontrolled or difficult-to-control COPD or asthma; multiple exacerbations; or recurrent respiratory tract infections^22^. Finally, the presence of allergic bronchopulmonary aspergillosis (a condition also usually associated with bronchiectasis) should be ruled out in patients with uncontrolled asthma^23^.

## Diagnostic methods

**Fig. 1:**
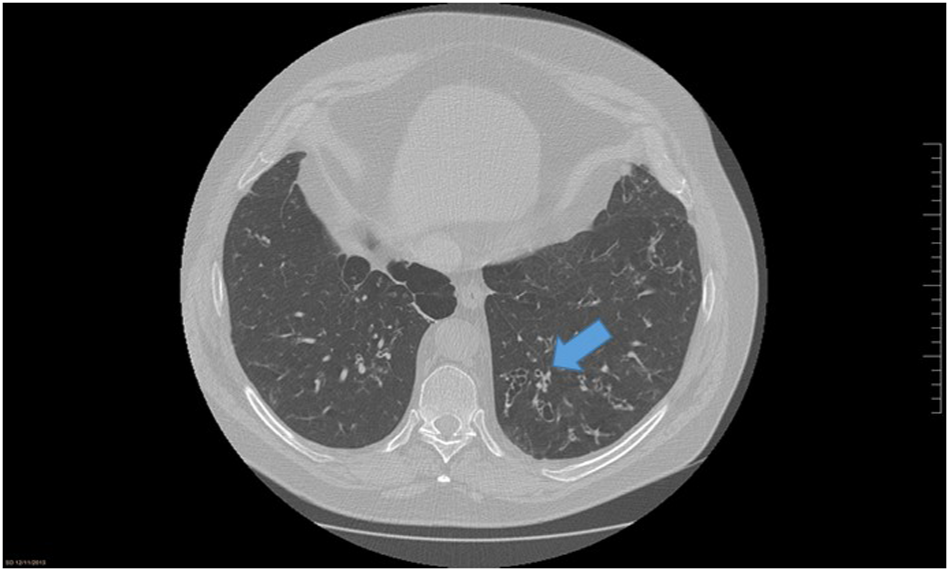
CT Ring sign in bronchiectasis. The “ring sign” is the most widely used radiological criteria for the radiological diagnosis of bronchiectasis. It is defined as a luminal diameter greater than that of the accompanying pulmonary artery.

Bronchiectasis is not visible in a simple chest X-ray, except in a few severe cases, so a *high-resolution computed tomography (HRCT)* is needed for its diagnosis. The diagnosis should therefore be made by a secondary/tertiary centre where HRCT scan is available. The most widely used radiological criteria for this diagnosis are: bronchial dilation (a luminal diameter greater than that of the accompanying pulmonary artery called “ring sign”, Fig. 1); lack of bronchial tapering; and dilated bronchi close to the pleura. Bronchial wall thickening may also be present as a marker of bronchial inflammation^24^. It is important to stress, however, that the radiological diagnosis of bronchiectasis can produce false positives (hypoxic vasoconstriction in chronic respiratory diseases), and false negatives (pulmonary hypertension due to the increased diameter of the pulmonary vessel). Furthermore, bronchiectasis can sometimes be reversible, especially in children after infectious processes (pseudobronchiectasis), and dilated bronchi can be seen in the elderly and people living at high altitude^25^.

Furthermore, it must be highlighted that the diagnosis of bronchiectasis is clinical-radiological, i.e., apart from characteristic radiological images, there must also be a clinical picture associated with bronchiectasis—usually productive cough (normally with a purulent component) and exacerbations with an infectious profile^11^.

## Severity assessment

Recently, three predictive multidimensional scoring systems, the BSI (*Bronchiectasis Severity Index*)^26^, the FACED^27^ and the E-FACED^28^ scores have been developed and validated to assess disease severity in bronchiectasis. FACED and E-FACED are simpler and easier to use (5 and 6 variables, respectively) and have an excellent predictive value for mortality (FACED and E-FACED) and future exacerbations/hospitalizations (E-FACED). The BSI was originally constructed and validated against more important outcomes in bronchiectasis including mortality, hospital admissions, exacerbations and quality of life, although it is composed of 9 variables. These scores could help to identify high-risk patients (more severe disease, higher risk of frequent exacerbations, hospitalization or mortality) who may benefit from a coordinated follow-up with tertiary care. Table 1 shows the composition of these scores and classification as mild, moderate and severe bronchiectasis based on them. Finally the *Bronchiectasis Aetiogy and Comobidity Index* (BACI)^29^ score was developed to quantify the impact of common comorbidities, therefore this index could also be used to identify high-risk patients who also might need coordination of primary and secondary/tertiary care.

**Table 1:** Multidimensional scoring systems^a^.

A. BSI score			
Severity marker	Score points	Severity marker^b^	Score points
*Age*		*Previous exacerbations*	
<50	0	0	0
50–69	2	1–2	0
70–79	4	3 or more	2
80 or more	6		
*Body mass index (kg/m*^*2*^*)*		*MRC dyspnoea score*	
<18.5	2	1–3	0
18.5–25	0	4	2
26–29	0	5	3
30 or more	0		
*FEV1 % predicted*		*Pseudomonas aeruginosa* *colonization*	
>80%	0		0
50–80%	1	No	3
30–49%	2	Yes	
<30%	3		
*Previous hospital admissions*		*Colonization by other pathogenic microorganisms*	
No	0	Yes	0
Yes	5	No	1
*Radiological severity*			
No	0		
Yes	1		

B. FACED and E-FACED scores^c^		
Variable	Values	Points
*F*ev_1_	At least 50%	0
	<50%	2
*A*ge	<70 years	0
	At least 70 years	2
*C*hronic colonization by PA	No	0
	Yes	1
*E*xtension (no. of lobes)	1–2 lobes	0
	More than 2 lobes	1
*D*yspnoea (mMRC)	0–II	0
	III–IV	1
	*Range:* 0–7 points	

^a^Severity bronchiectasis range: Mild (0–4 points); moderate (5–8 points), and severe (at least 9 points).

^b^Severity bronchiectasis range: Mild (0–3 points E-FACED; 0–2 points FACED); moderate (4–6 points E-FACED; 3–4 points FACED); and severe (7–9 points E-FACED and 5–7 points FACED).

^c^E-FACED also include (E) Hospitalization in the previous year (No 0 points; at least 1 = 2 points).

## Copd, asthma and bronchiectasis frequently coexist

**Fig. 2:**
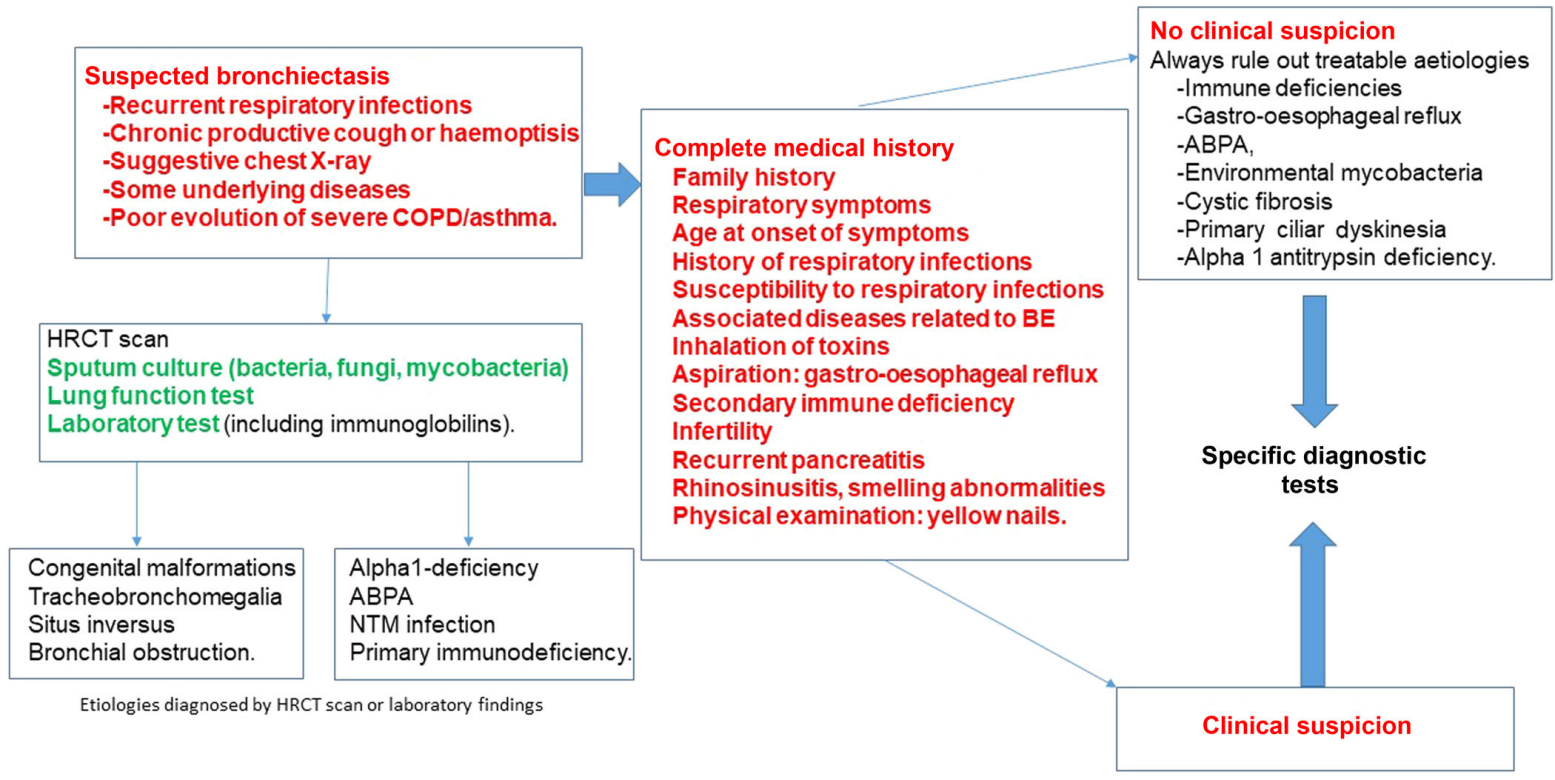
From suspected bronchiectasis to specific diagnosis test. In red: To be performed in Primary Care. In green: To be performed in Primary Care (if available). In black: To be performed in secondary/specialized centre. HRCT high-resolution computed tomography, COPD chronic obstructive pulmonary disease, ABPA allergic bronchopulmonary aspergillosis, NTM non-tuberculous mycobacteria, BE bronchiectasis.

A complete clinical history is essential in Primary Care to evaluate the most probable aetiology of bronchiectasis, especially when this is a treatable disease (Fig. 2)^13–15^. Sometimes the aetiological diagnosis is already known, and sometimes the information provided by an electronic clinical history or patients themselves gives the Primary Care physician some important clues, such as previous pulmonary infections of viral origin, pneumonias, tuberculosis or frequent rhinosinusitis or otitis. In any case, *although some tests should be performed and interpreted by the reference centre to rule out a specific aetiology, a coordinated approach between Primary Care and specialist/tertiary care is always required.*

It is important to bear in mind that the presence of bronchiectasis does not exclude the possibility of coexisting COPD or asthma. In fact, bronchiectasis is closely related to these two diseases, since it can appear in up to 35–50% of severe cases of COPD^30^ and in 25–40% of severe cases of asthma^31^. In both these situations, bronchiectasis increases the severity of the underlying disease and makes it more difficult to control. There is no definitive evidence that asthma and COPD are direct causes of bronchiectasis, although the available data suggest that bronchiectasis can form part of the natural history of both diseases, especially in their more advanced stages^32^. These conditions are known as *bronchiectasis-COPD (BCOS)* and *bronchiectasis-asthma (BAOS) overlap syndromes*, and treatment should aim to handle the two-component diseases separately, following the current guidelines^13–15^. However, one important point should be highlighted: since inhaled corticosteroids (ICs) are not recommended in bronchiectasis, the general recommendation is that ICs should be used in BCOS or BAOS only when indicated, following the guidelines for asthma or COPD. The lowest possible dose of ICs should be prescribed and every effort must be made to maximize bronchodilation—always keeping in mind the balance between risk (increased risk of infections) and benefit (anti-inflammatory properties) in the use of ICs^13–15^.

## Microbiological aspects

Although there is no clear consensus on this point, once bronchiectasis is diagnosed, it should generally be followed up in secondary care, or in specialized units in the most severe or difficult cases. *The role of Primary Care in the management of clinically stable patients is absolutely crucial, however, as is cooperation with tertiary care in severe cases*^21,33^. Other important factors are microbiological monitoring and serial sputum examinations (whenever possible). In the event of any clinical deterioration, changes in the appearance of the sputum (increased volume, density or purulence) or increased exacerbations, microbiological analysis of the sputum should be requested. The secondary centre should be contacted whenever pathogenic bacteria (including mycobacteria) are present, most particularly in the case of PA, since this microorganism has been shown to most significantly affect a patient’s prognosis^34^. The persistence of the same microorganism in the respiratory samples of a patient with bronchiectasis is called *chronic bronchial infection*^11,35^, and it should be managed in a secondary or specialized centre especially in clinically severe cases or significant comorbidities. Any isolation of fungi such as *Candida spp* or *Aspergillus* spp. should be considered colonization unless it is accompanied by a clinical picture suggesting an active infection that should be treated^13–15^.

## Therapeutic aspects

**Fig. 3:**
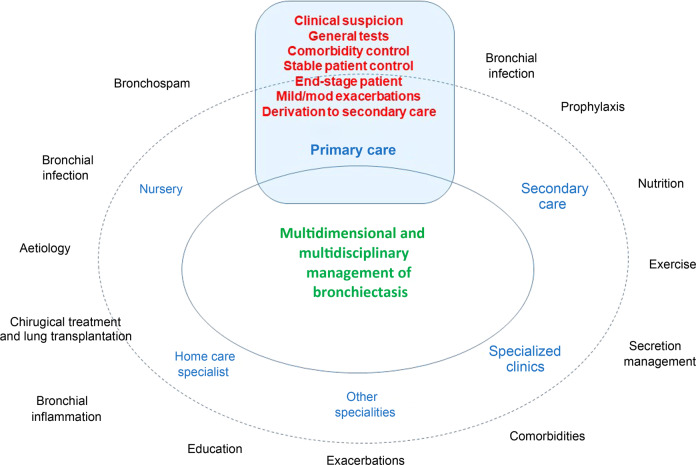
Multidimensional and multidisciplinary management of bronchiectasis. Role of Primary Care. Bronchiectasis is a disease that is both multidimensional (since it is necessary to treat all its therapeutic aspects or treatable traits) and multidisciplinary (since various specialities must monitor these patients). Within this multidisciplinarity, the role of Primary Care is absolutely essential in many aspects such as clinical suspicion, the performance of diagnostic or aetiological tests, monitoring of stable patients, collaboration with secondary/tertiary care in complex patients, management of end-stage patients, management of exacerbations and comorbidities and referral of the patient to secondary/tertiary centres, when necessary.

**Fig. 4:**
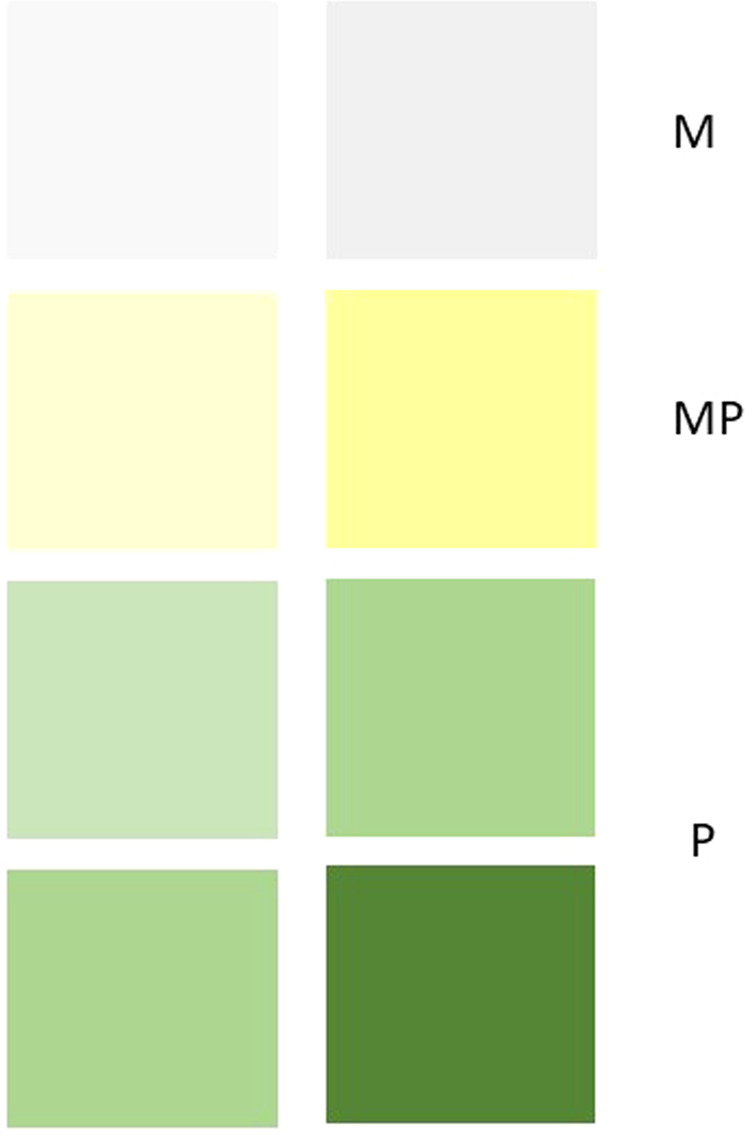
Table to assess the colour of sputum from least to most purulent. Murray´s colorimetric scale is a very simple tool to assess the inflammatory component of the sputum and the response to treatment. Modify from Murray et al. *Eur. Respir. J.* **34**, 361–364 (2009) (*Full free article. No permission is required)*: M mucous, MP mucopurulent, P purulent.

The diagnostic and therapeutic management of bronchiectasis is both multidisciplinary (pulmonologists, internists, nutritionists, endocrinologists, physiotherapists, radiologists, microbiologists, nursing staff and, of course, Primary Care teams) and multidimensional (treatment of the aetiology, infection, inflammation, exacerbations, complications, end-stage, etc.) (Fig. 3). Although there is no consensus on the characteristics of those patients who might benefit from different levels of care^36^, in general, stable patients with bronchiectasis can be managed in Primary Care. However, joint management with specialized units is recommended for patients with chronic bronchial infection by pathogenic microorganisms (particularly when associated with comorbidities with severe asthma and COPD)^36,37^, a lack of clinical stability, aetiologies that are difficult to manage^22,33^ or frequent exacerbations. Some practical advice for the management of patients with bronchiectasis in Primary Care could be as follows:

- It seems obvious that some general management points concur with those applicable to other chronic airway diseases: good education about the disease and about inhalation techniques; adequate vaccination against influenza, SARS-CoV-2 and *Streptococcus pneumoniae*; cessation of smoking; regular clinical visits; promotion of good health-related and dietary habits; and smooth coordination with the secondary/specialist centre if the patient needs to be referred^22,35,38^.
- Although numerous studies are underway on severity and therapeutic and prognostic biomarkers in bronchiectasis, sputum colour remains one of the simplest and most effective markers. Thus, one of the main objectives of treatment is to reduce the purulent component in the sputum as much as possible, following Murray’s colorimetric scale (Fig. 4)^39^.
- It is important for patients with bronchiectasis, especially those with expectoration, to follow *respiratory physiotherapy/rehabilitation* that can be facilitated from their Primary Care centres in conjunction with physiotherapeutic units, especially in those patients functionally limited by breathlessness. Some international guidelines strongly recommend that patients should be taught to carry out airway clearance techniques, such as an active cycle of breathing, or use an oscillating positive expiratory pressure device such as the “Flutter” and “Acapella”. These techniques are tailored to individual patients; they are usually performed once or twice a day, although this rate can be increased during an exacerbation. Moreover, airway clearance may be optimized by using postural drainage (gravity-assisted positioning to drain areas of the lung) and prior inhalation of isotonic or hypertonic saline^13–15^.
- Treatment with *inhaled corticosteroids should be avoided,* except in the case of the coexistence of asthma or COPD with eosinophilia, as established by the guidelines^13–15,40,41^. In any case, it is prudent to give patients the lowest possible doses, optimizing bronchodilation in the event of symptoms or chronic airflow obstruction.
- *Macrolides* are usually prescribed in bronchiectasis due to their immunomodulatory properties, so it is usual for this treatment to last for months, and for doses to be lower than usual (it is customary to use azithromycin 250–500 mg three times per week on non-consecutive days)^13–15^.
- One of the fundamental aspects that must be managed from Primary Care in patients with bronchiectasis associated with other chronic airway diseases (or, indeed, with chronic airway disease in general) is the *control of comorbidities*. These frequently include depression, anxiety^42,43^ and impairment in social aspects^44^, and they must be treated. Primary Care plays a fundamental role in this respect.
- It is important to become familiar with *inhaled antibiotics* and the different devices that are used, especially electronic or mesh devices. The role of nursing in this respect is fundamental^45,46^.
- The *treatment of mild-moderate exacerbations* not caused by PA can be managed in Primary Care. However, it is important to promptly inform the bronchiectasis unit of any severe exacerbations, or of any exacerbations caused by PA, since the treatment will generally have to be more aggressive^13–15^.
- The *management of an end-stage patient* with bronchiectasis does not differ, in general, from that of any chronic respiratory patient^13–15^.
- We cannot forget that after more than two years of the *SARS-COVID-19* pandemic, a high percentage of patients have suffered from chronic respiratory complications, including alterations to the bronchial tree such as traction bronchiectasis, with an evolution that cannot currently be predicted^47^. Primary Care plays a fundamental role in referring those patients who present, after suffering a pneumonic process (especially in severe forms), exacerbations of the infectious profile, isolation of pathogenic microorganisms or an increase in the quantity, density or purulence of sputum^48^.
- Finally, Primary Care must become familiar with new concepts and new technology, which has come a long way after the COVID-19 pandemic, as in the telemedicine control of patients (especially stable chronic patients, including those with bronchiectasis)^49,50^, and the use of devices^51^ and concepts such as the microbiome and its dysbiosis^52,53^ that are being introduced and promoted within medicine.

In short, the role of the Primary Care team is crucial in patients with bronchiectasis. It should focus, above all, on solid clinical suspicions, good communication with the reference centre, clear criteria for patient referral, thorough knowledge of the peculiarities of bronchiectasis that distinguish it from others such as COPD and asthma and management of stable and end-stage patients (Box 2).
